# Enabling data linkages for rare diseases in a resilient environment with the SERDIF framework

**DOI:** 10.1038/s41746-024-01267-6

**Published:** 2024-10-04

**Authors:** Albert Navarro-Gallinad, Fabrizio Orlandi, Jennifer Scott, Enock Havyarimana, Neil Basu, Mark A. Little, Declan O’Sullivan

**Affiliations:** 1 ADAPT Centre for Digital Content, School of Computer Science and Statistics, Trinity College Dublin, Dublin, Ireland; 2 Health Data Science Centre, Fondazione Human Technopole, Milan, Italy; 3 Trinity Kidney Centre, Trinity College Dublin, The University of Dublin, Trinity Translational Medicine Institute, Dublin, Ireland; 4 Institute of Infection, Immunity and Inflammation, College of Medical, Veterinary and Life Sciences, University of Glasgow, Glasgow, UK

**Keywords:** Scientific data, Public health, Vasculitis syndromes

## Abstract

Environmental factors amplified by climate change contribute significantly to the global burden of disease, disproportionately impacting vulnerable populations, such as individuals with rare diseases. Researchers require innovative, dynamic data linkage methods to enable the development of risk prediction models, particularly for diseases like vasculitis with unknown aetiology but potential environmental triggers. In response, we present the Semantic Environmental and Rare Disease Data Integration Framework (SERDIF). SERDIF was evaluated with researchers studying climate-related health hazards of vasculitis disease activity across European countries (*N*_P1_ = 10, *N*_P2_ = 17, *N*_P3_ = 23). Usability metrics consistently improved, indicating SERDIF’s effectiveness in linking complex environmental and health datasets. Furthermore, SERDIF-enabled epidemiologists to study environmental factors in a pregnancy cohort in Lombardy, showcasing its versatility beyond rare diseases. This framework offers for the first time a user-friendly, FAIR-compliant design for environment-health data linkage with export capabilities enabling data analysis to mitigate health risks posed by climate change.

## Introduction

Environmental exposures amplified by climate change can have a significant and increasing impact on human health that extends across geographical, cultural, and socioeconomic boundaries, contributing to a global burden of 23% of disability-adjusted life years (DALYs)^1,2^. The exposures’ impact varies across and within countries, populations and individuals due to their variety of genetic baselines, magnitudes of exposure and windows of susceptibility^3–6^. For example, early life exposures can result in short-term effects on health or accumulate and promote the development of diseases in later stages of life^7,8^. However, epidemiological studies that aim to predict and mitigate climate change impacts on public health need to overcome a complex data linkage process, where patient events are tracked through both location and time, linking key information in environmental and health datasets are linked^9–11^. Linking data is particularly challenging in rare disease research due to the need to connect geospatially sparse health events over a wide area (e.g. multiple counties, regions or countries) from heterogeneous data sources^12,13^. This is necessary to overcome the challenges posed by the low individual prevalence and the limited information available for each disease. In addition, the lack of shared unique identifiers between clinical and environmental datasets introduces technical complexities that require a linkage model to reconcile the differences in temporal and spatial granularity. Researchers need a clear provenance record to accurately integrate diverse datasets to achieve sufficient sample sizes for analysis and ensure the results are interpretable. This record is essential when multiple people work on the integrated dataset, serving as the key reference for everyone involved. This is the case of EU Horizon 2020-funded projects like the HEalth data LInkage for ClinicAL Benefit (HELICAL) project, where datasets within an interdisciplinary consortium need to be linked to gain new biological insights for autoimmune vasculitides^14^. For these reasons, environmental health studies will benefit from effective data linkage approaches to generate linked datasets that are usable in a research context.

The challenge of achieving effective data linkages has been successfully addressed in other domains, such as enterprise, retail, bioinformatics, biology, life sciences, public sector, etc.—as Hogan, A. 2020 nicely summarised in his article, by using a Knowledge Graph (KG) approach based on Semantic Web technologies^15^. This graph approach provides a harmonised data model with semantics making the data understandable and usable for both humans and software agents working on their behalf while enhancing efficiency, transparency and provenance when establishing new data linkages. In the context of environmental health, the application of KG approaches presents a novel frontier, where the development of a usable framework to guide the implementation of these technologies for health data researchers could reduce the technical complexities that are typically associated with data linkage tasks.

In this paper, we present a framework to enable health data researchers to efficiently link environmental and health data sources through location and time information, named the Semantic Environmental and Rare Disease Data Integration Framework (SERDIF). The proposed framework enables researchers to establish new links and construct datasets ready to be used for analysis and published following open science best practices, in line with the FAIR principles of Findability, Accessibility, Interoperability and Reusability (FAIR)^16^. As such, SERDIF is presented here as an interoperable, usable and open framework that can support health research that involves environmental perspectives in a manner that will support new insights for public health. This framework addresses the rare disease-specific challenge of ensuring clarity when linking data from multiple sources with different temporal and spatial units by maintaining a provenance record accessible for both machines and humans. This paper focuses on the final version of the SERDIF framework, shaped by the input of researchers studying paediatric and adult vasculitis in multiple countries within Europe.

## Results

To aid clarity of presentation of results, this section is structured following the four steps of a user-centred design^17^: a description of how the users may use SERDIF for their research (*context of use*), the identification of the expert user requirements (*expert user requirements*), the development of a refined framework for data linkage based on expert requirements (*data linkage framework*), and the usability testing results as evidence for the achievement of the requirements (*evaluation against requirements*). Then, validation use cases using real-world data are presented as successful stories, showcasing the practical application and effectiveness of the proposed approach compared with traditional methods.

### Context of use

Health and environmental data are complex within their own domain, but the complexity can be even higher for domain experts when linking these diverse types of data. The common data elements between health and environmental observations are time and location information. However, no direct relationship exists unless the temporal resolution, collected datasets, and geospatial area match precisely. Flexible linkages, informed by domain experts, are essential between datasets to support specific analyses in each use case. The complexity of the initial data sources related to the use cases (P1, P2 and P3) that required an effective data linkage method is highlighted in Table 1. In addition, each of the data sources in this table was accompanied by a set of metadata information that is not included in the table.

**Table 1:** Summary of the initial data sources to highlight the complexity of the data used in this paper for the 2011-2021 period

Data type	Format	Size	Variability	Structure	Temporal resolution	Spatial coverage	Phase
Disease registry	Relational database, RDF	16 MB	668 patients	Patient records	Daily	Ireland	1
National survey	CSV	2 MB	48 regions	Single location time series	Daily	Japan	2
Disease registry	Relational database, RDF	33 MB	1391 patients	Patient records	Daily	Ireland, UK, Switzerland, Czech Republic	3
Weather	NetCDF, HTML, TXT	1 GB	9 variables	Spatial grid time series	Daily	Europe	1–3
Air pollution	CSV	32 GB	462 variables	Single location time series	Daily, monthly, yearly	Ireland, UK, Switzerland, Czech Republic	1–3
Administrative areas	RDF	2 MB	1782 geometries	Nested geometries	_	Europe	1–3

### Expert user requirements

The initial set of expert user requirements was gathered from research meetings and through undertaking a consensus process with health data researchers in a preliminary study^18^. The initial requirements were refined in an iterative manner over the three phases of the usability study (P1, P2 and P3), which resulted in the following requirements.

*Requirement 1*: Enable health data researchers to query environmental data associated with relevant/own individual health events through location and time, within the area of the event and a period of data before the event.

*Requirement 2*: Support the understanding of event-environmental linked data and metadata, with its use, limitations, and data protection risk for individuals, by using a simplified view focused on the data linkage process with optional further information.

*Requirement 3*: Export event-environmental linked (meta)data to be used as input in statistical models for data analysis (CSV) and for publication (CSV, RDF).

### SERDIF data linkage framework

**Fig. 1:**
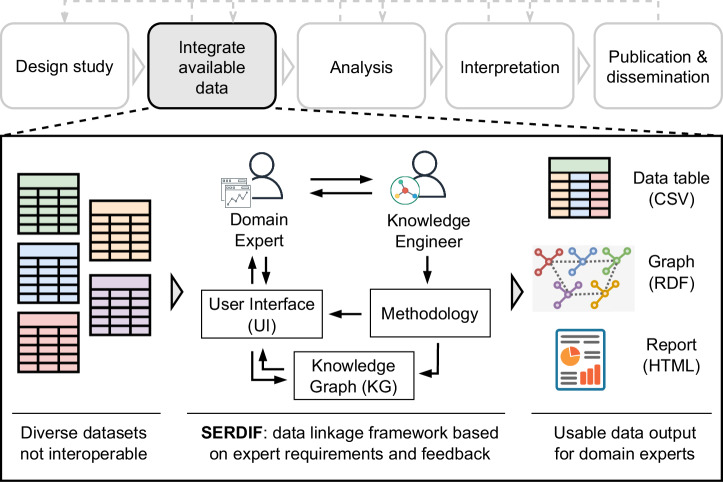
Role of SERDIF in researcher’s health-environment workflow. This figure depicts the integration of available data using SERDIF, positioned between study design and data analysis. It highlights how SERDIF links datasets to facilitate comprehensive analysis in the health-environment workflow.

The framework was designed to facilitate the data integration step for researchers in health-environmental workflows (Fig. 1). The framework is a combination of tools and processes to enable researchers to effectively link health and environmental data using a KG approach. In this context, “enable effective data linkage” refers to providing the means for researchers to (a) make their datasets interoperable, (b) create queries to link datasets to a particular event based on the spatial and temporal aspects of the data, and (c) export a transformed view of the linked data in a usable format for humans and machines.

The framework has three components: methodology, Knowledge Graph (KG), and User Interface (UI). The Methodology component describes a series of steps to facilitate the process of making data interoperable and usable for researchers while requiring and promoting the collaboration between domain experts and knowledge engineers. The SERDIF methodology steps from a technology-independent perspective and guidance for each of the methodology steps is provided based on the experience of the authors during the implementation of SERDIF (Table 2).

**Table 2:** SERDIF methodology technology independent steps and implementation annotated with design choices based on World Wide Web standards (W3C), generally accepted methods (GAM) and usability evaluation results (UER)

Methodology steps	Technology independent step and design choices Implementation
Step 0: Preparation	Perform the design study phase in a health-environmental workflow, defining the strategy to answer a research question using empirical data. The design study phase also requires a clear definition for the health events relevant to the study, and the permission to process the health event’s location and date to link it with environmental data. Another important element is the definition of potential queries to help explore the research question. **GAM.** Include the KG approach to link environmental datasets and health events through potential personal information (event’s location and time) as part of the data processing strategy and compliance.
Step 1: Data collection	Gather the available environmental datasets relevant to the research question of the study. The datasets are expected to have spatial and temporal features, which are required for Step 3: Data linkage. **GAM.** Include dataset metadata with at least the information related to the dataset descriptors (e.g. licence, title, version, temporal and spatial information and structure of the dataset), data provenance (e.g. distribution and download url), data use, agents that downloaded the data (e.g. researcher, software and entity) and the definitions for the environmental variables, including the units and source of information; and geometry data for relevant study areas
Step 2: Semantic uplift	Design and execute rules on how to make the environmental datasets gathered in Step 1: Data collection interoperable. W3C. Define the uplift mapping using the Relational database to RDF Mapping Language (R2RML) using the RDF Data Cube vocabulary (QB) for the data, and Geographic Query Language for RDF Data (GeoSPARQL), PROV Ontology (PROV-O), Data Catalogue Vocabulary (DCAT) and Open Digital Rights Language (ODRL) for metadata (Supplementary Fig. 1). **W3C.** Uplift environmental datasets to RDF graphs. W3C. Store the RDF files resulting from the execution of the mapping and the semantic (meta)data vocabularies in a triplestore with GeoSPARQL support
Step 3: Data linkage	Define a query template that links the environmental datasets within an area relevant to an event location and selects a period of data before that event date. The query template has placeholders (or variables) for users’ input (Step 4: Data visualisation) and should be designed to be generic enough to adapt to different data sources. **W3C.** Link datasets and events using a SPARQL query template with GeoSPARQL (spatial) and xsd:dateTime (temporal) reasoning functionalities to establish new relationships adequate for each use case (Supplementary Figure 2).
Step 4: Data interaction	Design an initial User Interface (UI) to allow non- technical users to (i) input the minimum event data required to link with environmental datasets, (ii) specify the user’s relevant data linkage variables for the query template defined in Step 3: Data linkage, and (iii) execute the data linkage query and export the linked data and metadata generated as a data table for analysis, a graph for publication and an interactive report for exploration. **UER.** Design a simple UI on top of the KG focused on the data linkage process that allows for the input of health events with minimum information for the spatiotemporal linkage, the selection of linkage options and the export of linked data and metadata as a data table (CSV), graph (RDF) and interactive report (HTML).
Step 5: Usability evaluation	Evaluate the usability and potential usefulness of the UI solution defined in Step 4: Data interaction in achieving the user requirements. Conduct the evaluation in an iterative manner progressing from version to version until the user requirements are achieved. **GAM.** Combine summative and formative conceptualisations of usability as evidence for achieving the expert requirements and using standard usability metrics when possible.

The vocabularies and languages refer to W3C recommendations and standards using Semantic Web technologies.

The KG component addresses the data interoperability aspect by uplifting the tabular datasets to standard graphs, importing these graphs to a database, and exposing an API for researchers to run queries against the constructed KG. In particular, the W3C implementation of SERDIF (steps 2 and 3 in Table 2) structures data in RDF format with a standard subject–predicate–object triples representation of data (Box 1). Researchers can formulate SPARQL queries to link, retrieve and manipulate this RDF data, leveraging the flexibility and power of this query language to extract meaningful insights (Supplementary Fig. 1). In addition, the KG provides the means to explain the linkage process between health and environmental data based on researchers’ input, with a view to enhancing the use of the data within appropriate contexts.

**Fig. 2:**
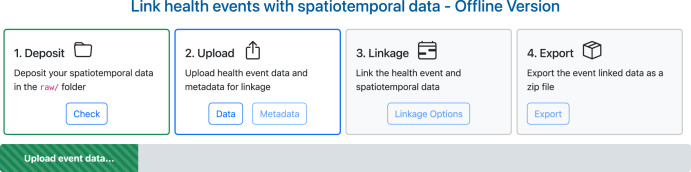
Screenshot of the SERDIF UI displaying the data linkage steps. This screenshot displays the SERDIF user interface with the four data linkage steps: (1) deposit environmental data, (2) upload health data, (3) define linkage options, and (4) export linked datasets.

The UI component is a tool towards addressing usability of interoperable data from the KG component (Fig. 2). The UI makes the query process intelligible for domain experts, the resulting linked data from the query easier to understand given a specific context of use and provides export functionality to retrieve the linked data for analysis and publication.

Box 1 Web standard knowledge graphs Semantic Web technologies refer to the World Wide Web Consortium (W3C) standards to support the Web of data with the goal of making data machine- and human-understandable^45^. The W3C developed the resource description framework (RDF) as the standard graph data model for data interchange and publication of information on the Web (i.e. standard-based implementation of knowledge graphs). RDF data is structured as a graph where real-world concepts are represented as subject–predicate–object statements (triples), capable of describing any type of data. The entities (subject) and relationships (predicate) of the statements are identified with Uniform Resource Identifiers (URI), and the object entity is either a URI or a literal (of type string, date, integer, etc.). RDF can use RDF schema (RDFS), the web ontology language (OWL) languages to describe groups of related resources and relationships between these resources and provide meaning (semantics) and logic rules understandable by machines as vocabularies and ontologies. The RDF SPARQL query language (SPARQL) can be used to express queries across diverse data sources in RDF with the capabilities of querying graph patterns for tasks such as data linkage, exploration, aggregation, transformation, annotation, or validation tasks, to name a few.

### Evaluation against requirements

SERDIF was evaluated with three real use cases that required meaningful data linkage of health and environmental data to validate hypotheses of environmental risk factors for rare diseases. The evaluation included a total of 30 different health data researchers studying adult and paediatric vasculitides, ANCA-Associated Vasculitis (AAV) in Ireland (P1) and Europe (P3), and Kawasaki Disease (KD) in Japan (P2). The usability study was able to discover >90% of the usability problems that can happen 10% of the time, given the last pool of researchers (*N*_P3_ = 23)^19^. Researchers were asked to complete a series of data linkage tasks, refined throughout the three phases of the study following a think-aloud protocol while interacting with the SERDIF UI.

**Fig. 3:**
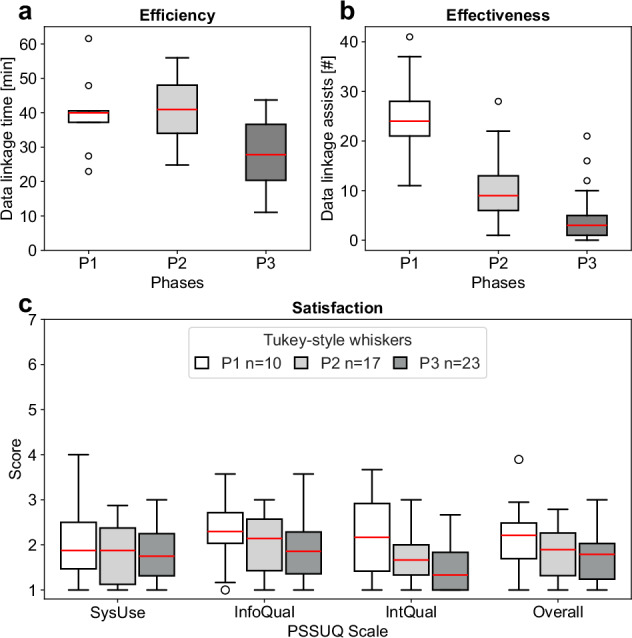
Progression of the comparable quantitative metrics for usability collected in the Phase 1, 2 and 3 of the evaluation. The participants denoted by *n* refer to the number of health data researchers that participated in each of the evaluation phases (P1, white, *n* = 10; P2, grey, *n* = 17; P3, dark grey, *n* = 23). **a** Efficiency**:** time spent per participant during the data linkage tasks. **b** Effectiveness**:** number of assists from the moderator during the data linkage tasks. **c** Satisfaction**:** Post-Study System Usability Questionnaire (PSSUQ v2) results for the System Usefulness (SysUse), Information Quality (InfoQual), Interface Quality (IntQual) and Overall scales. The lower the value of the quantitative metrics, the higher the usability.

The progress of usability metrics (efficiency, effectiveness, and satisfaction) across the three phases (P1–P3) is presented in Fig. 3. The efficiency improved from a median of 40 to 28 min between P2 and P3 for the data linkage tasks (Fig. 3A). The effectiveness improved once more in Phase 3 as denoted by the lower number of assists required per participants when completing the data linkage tasks (Fig. 3B). The satisfaction identified from the PSSUQ scales improved slightly from P2 (Fig. 3C). The usability problems decreased between phases as denoted by the themes and findings that emerged after conducting a thematic analysis that combined the quantitative and qualitative usability metrics (Supplementary Table 1). The potential usefulness of SERDIF increased across phases, with more findings associated with this metric. Beyond the qualitative progress based on the findings and references, the findings can be classified as usability problems or potential usefulness categories to quantify their decrease and increase, respectively. The decrease in usability problems results in P1 = 413 (73%), P2 = 1306 (68%) and P3 = 631 (39%) despite increasing and generalising the pool of researchers. The increase in potential usefulness results in P1 = 126 (23%), P2 = 624 (32%) and P3 = 985 (61%) (Supplementary Table 1).

### Validation cases using real-world data

SERDIF was validated in three studies investigating the environmental factors associated with (1) systemic vasculitides in the UK, (2) AAV in Ireland and the UK, and (3) preterm birth in Lombardy (Italy).

1. *Systemic vasculitides in the UK*: Researchers explored the possibility of linking environmental data (air pollution and weather data) with the UK vasculitis registry. The aim of this linkage was to investigate the short- and long-term effect of historical weather patterns (temperature, wind and humidity) on the incidence of vasculitis. SERDIF was used to validate current traditional linkage methods towards improving the efficiency in linking historic environmental data with patient data. A key finding from this comparison was that SERDIF showed to be faster and more robust in linking both the weather and air pollution data through internal nearest neighbour stations using intersecting and proximity polygons^20^. The linked datasets were ready to be used for the development of disease predictive models to predict the impact of climate change (e.g. distributed lag non-linear models), and more specifically effects of previous weather patterns on disease incidence^21,22^. Figure 4A presents an example of the type of modelling outputs used to predict the lagged effect of weather variables on rare disease incidence, as applied to the vasculitis registry in the UK. Though the analyses were exploratory and the findings are not generalisable, they show what’s possible when appropriate and effective linkage tools and methodologies are applied to rare disease research.

**Fig. 4:**
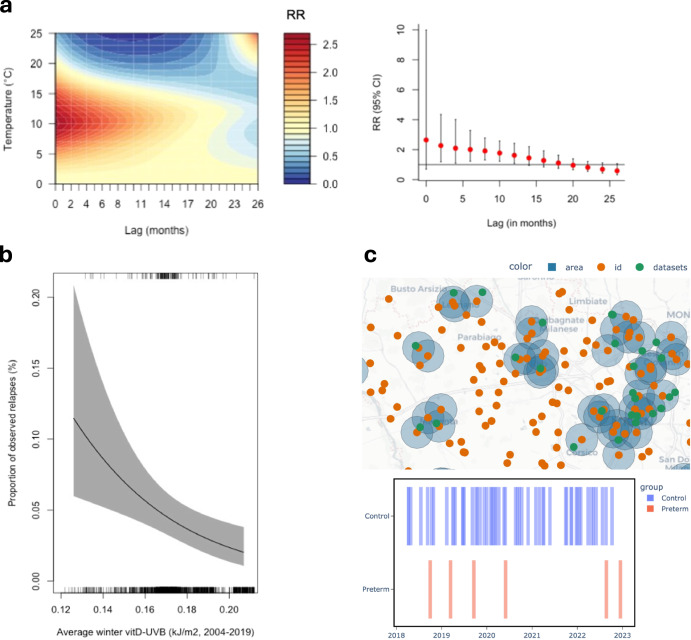
Examples of how data linked through SERDIF can be used to model and predict environmental exposures associated with rare disease incidence and relapse and visualise the spatiotemporal linkage process for pregnancy cohorts. **a** The UK vasculitis registry was used to evaluate the lag-response association between historical weather patterns and the incidence of AAV. These figures were generated on a small subset of the vasculitis registry and cannot be generalised to the vasculitis population in the UK. **b** Effects plot demonstrating the marginal effect of average winter vitD-UVB (kJ/m2) (UVB at wavelengths specific for vitamin D synthesis) on relapse risk in Ireland. We demonstrated that a patient’s specific exposure (i.e. low ambient UVB, particularly during winter), is associated with an increased risk of relapse in AAV. **c** Interactive visualisation (html) of small-area linkages for specific time windows, for example, pregnancy events and real environmental data in Lombardy.
2. *AAV in Ireland and the UK*: Researchers investigated the effect of ultraviolet (UV) radiation and vitamin D on AAV relapse risk in Ireland, using an *n*-of-1 study design^23^. Individual-patient level UVB dose, captured at the highest spatial and temporal resolution to date, was modelled. This required dynamic spatiotemporal linkage of UVB data with health data from the Irish Rare Kidney Disease registry (Table 1) as the patient moved through space and time over their disease course. The original linkage process applied in this study was complex and labour-intensive. This was subsequently successfully replicated using SERDIF. Similar to its application in the UK (above), SERDIF was faster, more efficient and more user-friendly, while maintaining accuracy. Figure 4B exemplifies the effect model output to demonstrate how a patient-specific exposure can be associated with an increased risk of relapse after an effective linkage process. SERDIF is currently being leveraged to explore the effect of other climate-related health hazards (including weather and pollution variables) on AAV risk in Ireland, using a multi-modal approach.
3. *Preterm birth in Lombardy*: Researchers were able to conduct small area-level linkages of layers of environmental observations to population-based data on health outcomes in pregnancy and at birth from administrative health records (~70k births per year, 2012–2023) in Lombardy. This spatiotemporal linkage is enabling the identification of environmental causes of preterm births (<37 weeks gestation), assumed to be the main drivers of its unclear aetiology^24^. The preliminary results of this case study were presented at the 12th Congress of the Società Italiana di Statistica Medica ed Epidemiologia Clinica (SISMEC) in 2023 (Fig. 4C).

Overall, researchers were able to efficiently link relevant weather and pollution datasets from local environmental stations and satellites with individual onset and relapse events of AAV patients from multiple patient registries, and with pregnancy events from administrative data at a regional level. Furthermore, SERDIF enabled the definition of a window of exposure to study a period of increased vulnerability for the health of AAV patients and pregnant women. The resulting linked exposure dataset for both projects included an interoperable distribution (RDF) ready to be deposited in an open data repository towards making the dataset FAIR comply with open science practices. An example of the resulting FAIR datasets from the studies is made available in Zenodo, including an HTML report (Fig. 4C), RDF data and metadata graphs, and CSV datasets at different aggregation levels^25^.

## Discussion

The usability study yielded a satisfactory outcome for SERDIF in meeting the expert user requirements for efficiently linking health events and environmental data in a rare disease context. This context required a usable, interoperable and open data framework to support rare disease research on climate resilience.

The interoperability aspect of the framework is given by using Web standard knowledge graphs (Box 1), making the graph data model understandable for machines with shared semantics and standards. Semantics provide enough context for machines to effectively interpret the intended meaning of the data. This contributes to higher levels of interoperability and scalability, addressing the most complex step for researchers in making data FAIR^26^. This poses an advantage in data-rich disciplines like the health-environment, enabling machines to efficiently perform tasks beyond human capability. Furthermore, the graph data model provided flexibility by emphasising the generation of new relationships between data points, fostering novel insights from existing data. This flexibility enables more complex queries to be used for scientific research by generating query templates that can infer relationships between health events and environmental data based on location and time without a shared identifier.

The usability aspect of the framework was evaluated in a three-phase study, with each phase involving the refinement of the expert user requirements and the SERDIF components. The summative usability part of the evaluation provided evidence for the improvement of the usability (efficiency, effectiveness, and satisfaction) of SERDIF against previous Phases (Fig. 3). The formative usability part of the evaluation (observational findings) supported SERDIF in being usable for non-technical users and potentially useful for health-environment researchers (Supplementary Table 1). However, the combination of some important data linkage features and text descriptions being unclear and the lack of preparation from the participants required guidance from the moderator to complete the tasks. The mixed methods approach, combining thematic analysis with quantitative metrics to support the findings and the transparency of the reporting of these findings, provided an effective means to refine the framework efficiently. The usability improvement throughout the study reinforces the adequacy of the evaluation approach taken to evaluate the framework against the three-user expert requirements and demonstrates the general applicability of the framework within different health and environmental data contexts.

The openness aspect of the framework is provided by following best practices in open data publication in line with the FAIR principles^16^. The SERDIF implementation based on W3C standards produced a linked data output as an RDF graph ready for publication. The RDF output also includes information about the origin of the dataset together with the processing steps to generate the dataset (i.e. provenance metadata). Then, the researcher can make FAIR by: (i) licensing the dataset, specifying the data use, and (ii) defining the accessibility of the dataset and metadata when deposited in an open data repository. The achievement of FAIR data practices and goals can benefit future European and International projects with data linkage tasks present in their agendas. These projects can follow the technology-independent design of the methodology component of SERDIF or adopt the W3C standard approach design choice based on their goals and context. An offline and ready-to-use distribution of SERDIF was made available to the community under the permissive and open-source MIT licence: https://w3id.org/serdif. This distribution would enable projects with personal and/or sensitive data to work offline with a semi-automatic process to effectively link the data based on user inputs. The offline distribution can only input environmental datasets in certain formats (.nc, .grid, .csv and .tsv) with specific column names, as indicated in the user interface, which was noted by researchers during the validation use cases with real-world data.

SERDIF’s design and usability were shaped to provide an efficient alternative to traditional data linkage methods for linking health and environmental data, leveraging meaningful feedback from health data researchers. These researchers, typically using ad-hoc approaches such as SQL, Python, or R scripts with geospatial libraries, found SERDIF’s knowledge graph (KG) approach to be a novel and efficient alternative. The usability study revealed that SERDIF offers significant advantages in terms of efficiency, effectiveness, and satisfaction compared to traditional methods, as evidenced by the feedback from both technical (i.e. data scientists or technicians) and higher-level conceptual researchers (i.e. clinicians, epidemiologists, and principal investigators). SERDIF enhances data linkage workflows with its user-friendly interface, efficient processing, flexible querying, and adherence to W3C standards. By using SPARQL and knowledge graphs, the framework offers tailored queries for specific research needs and standardises data linkage processes, ensuring consistency across projects with multiple partners and diverse data sources. Notably, state-of-the-art reviews highlight usability problems and structural heterogeneity as major challenges in integrating heterogeneous health data, recommending semantic web technologies like SERDIF to address these issues^27,28^. The validation of SERDIF in three real-world studies demonstrated its effectiveness in linking complex health and environmental data, supporting the generation of predictive models and enabling the identification of environmental influences on health outcomes.

The SERDIF framework requires researchers to define events with a date and location to be linked with spatiotemporal observations from environmental data. This affordable requirement makes the framework adaptable for studying the environmental interaction from various diseases and health events such as respiratory and cardiovascular diseases. In addition, SERDIF can potentially be applied a range of other fields, such as analysing environmental impacts on animal or plant populations (ecological), comparing survey perceptions with actual environmental conditions (sociological), highlighting environmental injustices (political), and documenting air quality changes based on industry emissions (sustainability). However, generalisability is limited by the spatiotemporal reasoning capabilities of W3C standards. While the standards cover temporal aspects such as intervals, lags and date manipulation, spatial linkage is limited to GeoSPARQL features^29^. Researchers should become familiar with GeoSPARQL capabilities during the *Preparation* step in the linkage process (Table 2). The implementation of SERDIF requires collaboration between health data researchers and KG experts to uplift the data to graphs and co-design the linkage query templates. The semantic uplift process and definition of SPARQL queries (Box 1) require technical expert knowledge to define the semantic models, execute the mappings and link the datasets through location and time (Supplementary Figs. 1 and 2). Despite the complexity associated with the implementation, the collaboration between health data and KG experts improves the flexibility of the input data involved and the validity of the linkage undertaken. This contrasts with other approaches in the health-environmental domain that use automated linking, ontology matching^30^ or interlinking approaches in this domain, but without guaranteeing authoritativeness^31,32^. A further limitation of this work is the narrow focus of the usability study, which only assessed SERDIF’s usability among health data researchers and did not include lay-users or extend to different tasks and contexts beyond environmental-health data linkage.

Future studies on more advanced use cases beyond ongoing rare disease studies can include logic rules to look for specific patterns across the data to discover new links in an automatic manner. Furthermore, the usability of the framework could be extended to patient cohorts, including data annotation tasks with relevant health-related information requested by a health professional in a self-reported manner. This information could be combined with existing health and environmental data for a more complete view of the health event. Beyond rare disease research, the framework has the potential to set the grounds for an early warning system to be used EU-wide. The early warning system would need to include an automatic data uplift stream to import the up-to-date and forecast data from the environmental sites and a connection with the healthcare centres to assess the health risk for specific vulnerable groups; and obtain the necessary approvals to be used as a risk assessment system in terms of GDPR and local regulations.

In conclusion, SERDIF is an effective framework for overcoming the complex challenges of linking environmental and health datasets when studying environmental triggers of vasculitis across Europe. The framework facilitates the data linkage process while generating an interoperable outcome ready to be shared as FAIR data. As SERDIF continues to gain traction in the research community, the use of this framework can lead to new insights for predicting and mitigating climate change impacts on public health that go beyond borders and disciplines, contributing to a better understanding of how the environment affects human health.

## Methods

### Study design and participants

The usability and potential usefulness of SERDIF were evaluated for a specific group of health data researchers (*expert users*) to conduct data linkage tasks (*goal*) towards studying the environmental factors associated with health events related to rare diseases (*context of use*). The study followed a user-centred design, considered a common approach to develop solutions for expert users^33^, to interact with KGs^34–36^. This approach consists of four iterative steps that continue until the solution meets the user requirements^37^:

1. The solution developer endeavours to understand the context of use by gathering insights about the domain, practical aspects, and real-world scenarios where the solution will be used.
2. Expert user requirements for the desired user interface are refined by collaborating with domain experts and users to expand upon an initial set of requirements.
3. A prototype solution is developed or refined based on the user's requirements.
4. Usability testing is conducted to evaluate the solution against the requirements.
  The user-centred design for SERDIF concluded after three progressive iterations, named Phase 1 (P1), Phase 2 (P2) and Phase 3 (P3), that included rare disease case studies that required meaningful data linkage to be undertaken by health data researchers, to test hypotheses of exploring environmental risk factors associated with health outcomes. The rare disease case studies included health data researchers studying ANCA-associated vasculitis (AAV) in Ireland (P1), Kawasaki disease (KD) in Japan (P2) and AAV in Europe (P3). AAV and KD are vasculitides that affect small and medium blood vessels in adults and children respectively, resulting in damage to vital organs. While the aetiology of these conditions is unknown, the current theory proposes a complex interaction between environmental and epigenetic factors in a genetically susceptible individual^38–40^. The participants in the study were health data researchers with a health background and statistical or data analysis experience, including clinicians, health data scientists and epidemiologists from academia and research institutes, and without practical expertise in using KG technologies. These researchers typically need to link patient registry data with weather and pollution datasets (P1, P3); and Japanese national survey data with aerosol datasets (P2). The use of SERDIF to support these rare disease case studies took place in a sequential and progressive manner, with the results from each case study informing the refinement of the requirements and framework before the next case study and increasing the pool of researchers from the previous phase (*N*_P1_ = 10, *N*_P2_ = 17 and *N*_P3_ = 23).
  The researchers participated in SERDIF usability testing sessions conducted remotely using the Zoom video conferencing platform, where participants were asked to share their screen and audio while observed and assisted, if necessary, by a usability moderator (authors of this paper). The recordings were manually transcribed to remove any personal information before storing the transcriptions in *Taguette*, a local database for coding text documents^41^. The participants were asked to complete a series of tasks during the sessions while following a think-aloud protocol^42^. The tasks were gathered and derived from consensus among researchers with real workflows in mind before the usability testing sessions. The tasks were the following:
  *Task 1*: Link environmental data to relevant (or example) health events for your research.
  *Task 2*: Export the data linkage output and explore the interactive report generated (.html).
  *Task 3*: Discuss if you are confident in using the linked data for your research.
  *Task 4*: Explain if you would need any additional features or information before starting the analysis of the linked data.
  *Task 5*: Summarise verbally your overall experience when linking data using SERDIF.

The evaluation approach was submitted to and gained approval from the relevant Ethics Committee in Trinity College Dublin (reference number: 20200201). Informed consent to participate in the study was obtained from all participants.

### Metrics

The three usability tests shared common elements to enable the comparison between phases such as the metrics gathered during the usability testing session, the data analysis methods, and instruments to evaluate the usability and potential of SERDIF based on researchers’ requirements^33^. The metrics combined quantitative (summative) and qualitative (formative) metrics to support the findings, following best practices^19^. The usability metrics were associated with the definition of usability (ISO 9241-11:2018) including the effectiveness, efficiency and satisfaction attributes, and the potential usefulness of the framework for health data researchers (ISO/IEC 25010:2011). The effectiveness was measured using the assists during task completion, as the moderator’s interventions during the Think-Aloud protocol can influence the task completion rates^42^. The efficiency was measured using the time spent in completed data linkage tasks (Tasks 1 and 2). The satisfaction was measured using the Post-Study System Usability Questionnaire (PSSUQ v2)^43^, a standard usability test survey, scores and scales gathered from the participants. The usability problems and potential usefulness of the framework were identified by the observational findings from the session transcripts.

### Data analysis

The metrics recorded during the usability testing sessions were analysed following the trustworthy thematic analysis approach by Nowell et al. ^44^, towards minimising the subjectivity and increasing the credibility of the qualitative analysis. The *Taguette* software^41^ and local database was used as a tool to facilitate the execution and documentation of the Thematic Analysis.

## Supplementary information

Supplementary material

## Data Availability

The datasets generated and/or analysed during the current study are available in the serdif repository, https://w3id.org/serdif.

## Code availability

The underlying code for this study is available in serdif and can be accessed via this link https://w3id.org/serdif.
