# Supplementary material for: Enabling data linkages for rare diseases in a resilient environment with the SERDIF framework
Source: NPJ Digit Med. 2024 Oct 4;7:274. doi: 10.1038/s41746-024-01267-6 (PMC11452697; doi:10.1038/s41746-024-01267-6)
Supplement: Supplementary file 1 — Supplementary material [file 41746_2024_1267_MOESM1_ESM.pdf]

Supplementary material

Supplementary Figure 1. SPARQL query template example for spatiotemporal reasoning in SERDIF. The query template includes namespaces, a query form clause, and a WHERE clause for filtering datasets based on event time intervals and geometries.

```
# -- 1. Namespaces -----------------------------------
PREFIX prov: <http://www.w3.org/ns/prov#>
PREFIX dcat: <http://www.w3.org/ns/dcat#>
PREFIX dct: <http://purl.org/dc/terms/>
PREFIX eg: <http://www.example.org/>
PREFIX xsd: <http://www.w3.org/2001/XMLSchema#> .

# -- 2. Query form clause -----------------------------
CONSTRUCT { ?event prov:used ?dataset . }

# -- 3. WHERE clause ----------------------------------
WHERE {

# -- 3.1 Event time interval and geometry ---------
?event a prov:Activity , geo:Feature ;
   prov:atLocation ?eventLoc ;
   prov:startedAtTime ?evStTime ;
   prov:endedAtTime ?evEndTime .

?eventLoc a geo:Geometry ;
   geo:asWKT ?eventGeo .

# -- 3.2 Time interval and geometry ----------------
?dataset a dcat:Dataset, geo:Feature ;
   locn:geometry ?datasetLoc ;
   dct:temporal ?datasetTemp .

?datasetTemp a dct:periodOfTime ;
   dcat:startDate ?dsStTime ;
   dcat:endDate ?dsEndTime .

?datasetLoc a geo:Geometry ;
   geo:asWKT ?datasetGeo .

# -- 3.3. Filter datasets within the geometry and time interval of the event –
# Geospatial reasoning
FILTER(geof:sfWithin(?datasetGeo, ?eventGeo))
# Temporal reasoning
FILTER(?dsStTime <= ?evStTime && ?dsEndTime >= ?evEndTime)
}

# -- Query results as RDF triples ----------------------
```

subject	predicate	object
eg:event-1	prov:used	eg:dataset-A
eg:event-2	prov:used	eg:dataset-A
eg:event-3	prov:used	eg:dataset-B

Supplementary Figure 2. Mapping used in SERDIF to uplift the environmental tabular data to an RDF graph written in R2RML. The mapping includes namespaces, a triple map for observation data, and SQL query examples for extracting and linking data on air pollutants, including definitions for time and pollutant name transformations.

```
# -- 1. Namespaces -----------------------------------
@prefix rr: <http://www.w3.org/ns/r2rml#> .
@prefix rrf: <http://kdeg.scss.tcd.ie/ns/rrf#> .
@prefix rdfs: <http://www.w3.org/2000/01/rdf-schema#> .
@prefix xsd: <http://www.w3.org/2001/XMLSchema#> .
@prefix sdmx-dimension: <http://purl.org/linked-data/sdmx/2009/dimension#> .
@prefix xsd: <http://www.w3.org/2001/XMLSchema#> .


# -- 2. Triple map for observation data -------------------------
<#MapObsData>

# -- 3. Select dates from datetimes -----------------------------
   rr:logicalTable [
       rr:sqlQuery """
SELECT CAST(DATETIMEBEGIN AS varchar(10)) AS TIMEG, AIRPOLLUTANT,
CAST(AVG(CAST(CONCENTRATION AS FLOAT)) AS DECIMAL (10,2)) AS CONCENTRATION
FROM {{data.eeaDataFile}}
GROUP BY TIMEG
       """;
    ];

# -- 4. Define observation (subject) based on lat, lon and date --
   rr:subjectMap [
        rr:template "https://serdif.adaptcentre.ie/kg/2022/dataset#type=airquality
&source=eea&version=vE1a&point={{data.lon}}_{{data.lat}}&time={TIMEG}";
        rr:termType rr:IRI;
        rr:class qb:Observation;
        rr:graphMap [ rr:template
"https://serdif.adaptcentre.ie/kg/2022/dataset#type=airquality&source=eea&version=vE1a&point={
{data.lon}}_{{data.lat}} "];
   ];

# -- 5. Link observations with dataset through location ----------
   rr:predicateObjectMap [
       rr:predicate qb:dataSet;
       rr:objectMap [
           rr:template
"https://serdif.adaptcentre.ie/kg/2022/dataset#type=airquality&source=eea&version=vE1a&point={
{data.lon}}_{{data.lat}}";
          rr:termType rr:IRI;
       ];
   ];

# -- 6. Define time dimension for each observaion ----------------
   rr:predicateObjectMap [
      rr:predicate sdmx-dimension:timePeriod ;
      rr:objectMap [
         rrf:functionCall [
            rrf:function <#time2datetime> ;
            rrf:parameterBindings (
               [ rr:column "TIMEG" ; ]
```

```
            );
         ];

# -- 7. Include air pollutant concentration values ---------------
   rr:predicateObjectMap [
      rr:predicateMap [
         rrf:functionCall [
            rrf:function <#pollutantNameClean> ;
               rrf:parameterBindings (
                  [ rr:column "AIRPOLLUTANT" ]
               ); ];
            rr:termType rr:IRI;
         ];
      rr:objectMap [
         rr:column "CONCENTRATION";
         rr:termType rr:Literal;
         rr:datatype xsd:float;
      ];
   ]; .

# -- 8. Define a function to format datetime values ------------
<#time2datetime>
   rrf:functionName "time2datetime" ;
   rrf:functionBody """
      function time2datetime(timeC) {
         // From 2010-01-01 to 2010-01-01T00:00:00
        return String(timeC ) + "T00:00:00" ;
      }
   """ ;
.

# -- 9. Define a function to clean pollutant names -------------
<#pollutantNameClean>
   rrf:functionName "pollutantnameclean" ;
   rrf:functionBody """
       function pollutantnameclean(pName) {
       // Fix format pollutant name to comply with URI standard symbols
      // by replacing parentheses, dashes, plus signs and commasdate time to conform with
standards
      // "Indeno-(1,2,3-cd)pyrene in PM10" -> "Indeno123cdpyreneinPM10"
      var pNameC = pName.replace(/[{()}]/g, '').replace(/\-/g, '').replace(/\+/g, '').replace(/,/g,
'').replace(/\s/g, '').replace(/\=/g, '') ;
      return "https://serdif.adaptcentre.ie/kg/2022/measure#has" + pNameC + "Value";
      }
   """ ;
.
```

Supplementary Table 1. Thematic analysis of SERDIF usability. The progression of the thematic analysis findings is summarised in this table to highlight usability problems and potential usefulness of SERDIF across the usability study. It details each evaluation phase, the themes identified, supporting evidence for these themes, and the frequency of theme references by participants.

Phase	Themes	Findings	References
P1	Useful approach for linking data	Positive overall user experience emphasising the data exploration features and the usefulness of SERDIF	126
	Requirements refinement	The origin and processing of the linked data is unclear and the environmental data needs to be linked for a period prior to the flare events	69
	Complex text and features	Some of the plots are complex and the technical jargon makes text descriptions hard to understand	75
	Testing methodology	The task's wording, delays and control malfunctioning during the virtual experiment session reduced the overall usability of SERDIF	269
P2	Requirement 1: Querying	While querying environmental data associated with particular events was possible, the event concept and approach were complex, confusing the query process	386
	Requirement 2: Understanding	The metadata is enough to understand the provenance and lineage of the linked data and the visualisations are useful to explore the data but the content is complex and hard to navigate making it not user friendly	660
	Requirement 3: Exporting	Exporting the (meta)data is simple and useful to be used as input for analysis	201
	Emerging Requirements	Additional features and explanations together with simpler words would increase the usability of the framework	260
	Usability testing	Overall positive experience when using the dashboard but moderator interventions were needed due to system technical issues and task design	423
P3	Requirements achieved	Health data researchers can link health events and environmental data for their research using SERDIF	796
	Potential uptake	SERDIF can be applied for real use cases with tailored environ- mental data and features even for non-technical researchers	189
	Minor improvements	Some important features and text descriptions are not clear in the UI	429
	Testing methodology	The lack of preparation from the participants increased the need for moderator's guidance	202
